# Collagen–Alginate Composite Hydrogel: Application in Tissue Engineering and Biomedical Sciences

**DOI:** 10.3390/polym13111852

**Published:** 2021-06-02

**Authors:** Tingyu Hu, Amy C. Y. Lo

**Affiliations:** Department of Ophthalmology, Li Ka Shing Faculty of Medicine, The University of Hong Kong, Hong Kong, China; tingyuhu@connect.hku.hk

**Keywords:** collagen–alginate composite hydrogel, tissue regeneration, wound dressing, tissue engineering, encapsulated cell therapy

## Abstract

Alginate (ALG), a polysaccharide derived from brown seaweed, has been extensively investigated as a biomaterial not only in tissue engineering but also for numerous biomedical sciences owing to its wide availability, good compatibility, weak cytotoxicity, low cost, and ease of gelation. Nevertheless, alginate lacks cell-binding sites, limiting long-term cell survival and viability in 3D culture. Collagen (Col), a major component protein found in the extracellular matrix (ECM), exhibits excellent biocompatibility and weak immunogenicity. Furthermore, collagen contains cell-binding motifs, which facilitate cell attachment, interaction, and spreading, consequently maintaining cell viability and promoting cell proliferation. Recently, there has been a growing body of investigations into collagen-based hydrogel trying to overcome the poor mechanical properties of collagen. In particular, collagen–alginate composite (CAC) hydrogel has attracted much attention due to its excellent biocompatibility, gelling under mild conditions, low cytotoxicity, controllable mechanic properties, wider availability as well as ease of incorporation of other biomaterials and bioactive agents. This review aims to provide an overview of the properties of alginate and collagen. Moreover, the application of CAC hydrogel in tissue engineering and biomedical sciences is also discussed.

## 1. Introduction

The vital role of biomaterials in tissue engineering and biomedical application has attracted much attention in the past decades [1]. Research into biomaterials and their application holds great promise in the future. Synthetic polymers had been extensively studied in the early twentieth century due to their better performance and ease of manufacture compared to natural materials [2,3]. Recently, naturally derived biomaterials have regained considerable interest in material science due to their extracellular matrix (ECM)-like structure that highly recapitulates in vivo environment, inherent biocompatibility, and a limited chance of immune response and immune rejection compared to human-made materials [4,5].

Alginate is a natural polysaccharide derived from brown algae with favorable properties, including broader availability, cost-effectiveness, good biocompatibility, weak cytotoxicity as well as ease of gelation in the presence of divalent cations [6]. Moreover, alginate has unique properties in comparison with other materials including, (1) ability to provide an inert aqueous environment, (2) sol-gel transition under the mild condition that enables excellent retention of bioactive molecules and cell encapsulation, (3) controllable porosity allowing adjustable molecular diffusion, (4) biodegradability under physiological environment, and (5) structural similarity to ECM [7,8,9,10]. These properties allow the wide investigation of alginate in tissue engineering and biomedical sciences, including cell encapsulation, wound dressing, tissue regeneration, and pharmaceutical application [11]. It is well known that anchorage-dependent cell death is activated in the absence of extracellular matrix [12]. Nevertheless, alginate inherently lacks cell-binding sites, thus limiting its wider-range application. Alginate modified with Arg-Gly-Asp (RGD) peptides or in combination with other biomaterials has been the focus of research.

Collagen is the primary protein found in soft connective tissue and hard connective tissue. Moreover, 33% of the protein found in the human body is collagen [13,14]. To date, 29 types of collagen are identified in the animals [15]. According to its structure, physical location in the body, and chain binding, collagen can also be divided into eight families [15]. Collagen-based hydrogel has been widely studied in tissue engineering and biomedical sciences owing to its excellent biocompatibility and weak immunogenicity [15,16,17,18,19]. Moreover, collagen, which contains RGD peptides, highly recapitulates ECM in the human body [20,21]. However, the insufficient mechanical properties limit its extensive application. It is ubiquitous to incorporate other biomaterials such as alginate, chitosan, and hyaluronic acid into collagen to improve its mechanical properties.

CAC hydrogel can be cross-linked under mild conditions. Therefore, CAC hydrogel is widely investigated for cell encapsulation and drug delivery [22,23,24,25]. What is more, the combination of collagen and alginate provides cell-binding motifs and enhances the mechanical properties [26]. There is a growing body of investigations into CAC hydrogel and its application in tissue engineering and biomedical sciences, including tissue regeneration, wound dressing, encapsulated cell therapy (ECT), and tumor biology. The composite hydrogel has been developed as a matrix scaffold to deliver a range of bioactive agents, cell populations, or in the combination of cell and bioactive factor to the defect site in tissue engineering. The composite hydrogel is also attracting much research attention in biomedical sciences. Collagen–alginate wound dressings can provide and maintain a moist environment to the wound site, promote cell migration, differentiation, thereby accelerating wound healing. CAC hydrogel is also promising for tissue regeneration due to its ease of gelling under mild conditions, which enables retention of bioactive agents and cell encapsulation. Alginate can provide encapsulation power that limits the chance of cell leakage from the ECT device. In addition, collagen can improve cell viability and proliferation. Therefore, CAC hydrogel has been exploited as a potential platform in ECT. Moreover, the composite hydrogels with tunning stiffness and higher similarity to ECM can mimic in vivo tumor cell environment. Therefore, it also explores a new field for the study on tumor biology.

**Figure 1:**
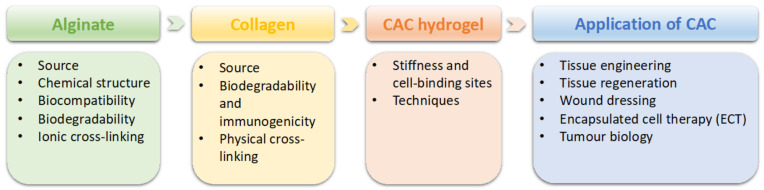
An overview of this review paper.

This review aims to provide a comprehensive review of CAC hydrogel and its application (Figure 1). Firstly, the characteristics of alginate and collagen will be given respectively, followed by presenting the properties of CAC and the techniques. Finally, the application of CAC in tissue engineering and biomedical sciences, especially tissue regeneration, wound dressing and encapsulated cell therapy, and tumor biology, will be described.

## 2. General Properties of Alginate and Collagen

### 2.1. Alginate

#### 2.1.1. Alginate Sources

Alginate can be obtained from two major sources, including brown seaweed and bacteria [27]. Three species of brown seaweeds, including *Laminaria Hyperborea*, *Ascophyllum Nodosum*, and *Macrocystis Pyrifera*, are the primary source for commercially available alginate [28]. Alginate can also be isolated from other algae species, including *Laminaria Japonica*, *Eclonia Maxima*, *Lesonia Negrescens*, and *Sargassum* [28]. The isolation of alginate from brown seaweeds requires multiple steps and procedures. With the exception of *Macrocystis Pyrifera*, which is processed in wet form, brown seaweeds are first cropped and dried for further extraction. In brief, the initial removal of unwanted materials and impurities from algae is achieved by addition of dilute mineral acid [29]. The preparation of sodium alginate is done by addition of alkali solution such as NaOH, followed by centrifugation to remove sediments. The removal of homopolysaccharides is done by adding mineral acid, ethanol, or CaCl_2_. After these procedures, there are remaining impurities in alginates, which require further purification in order to be used in the pharmaceutic field and biomedical sciences.

Alginate can be secreted by some bacteria, including *Azotobacter Vinerlandii* and *Pseudomonas* species. The synthesis of alginate by these two bacteria is highly similar. Alginate synthesized by bacteria varies in mechanical properties and has been utilized for different applications. Alginate isolated from bacteria possesses more chemical structure and physical properties than brown algae sources. The biosynthesis of alginate by bacteria has already been illustrated. In brief, precursor substrate was firstly synthesized, followed by polymerization on the precursor. It was then transferred to the cytoplasmic membrane and periplasmic membrane for further modification, finally transported to the surroundings [30].

#### 2.1.2. Chemical Structure and Molecular Weight

**Figure 2:**
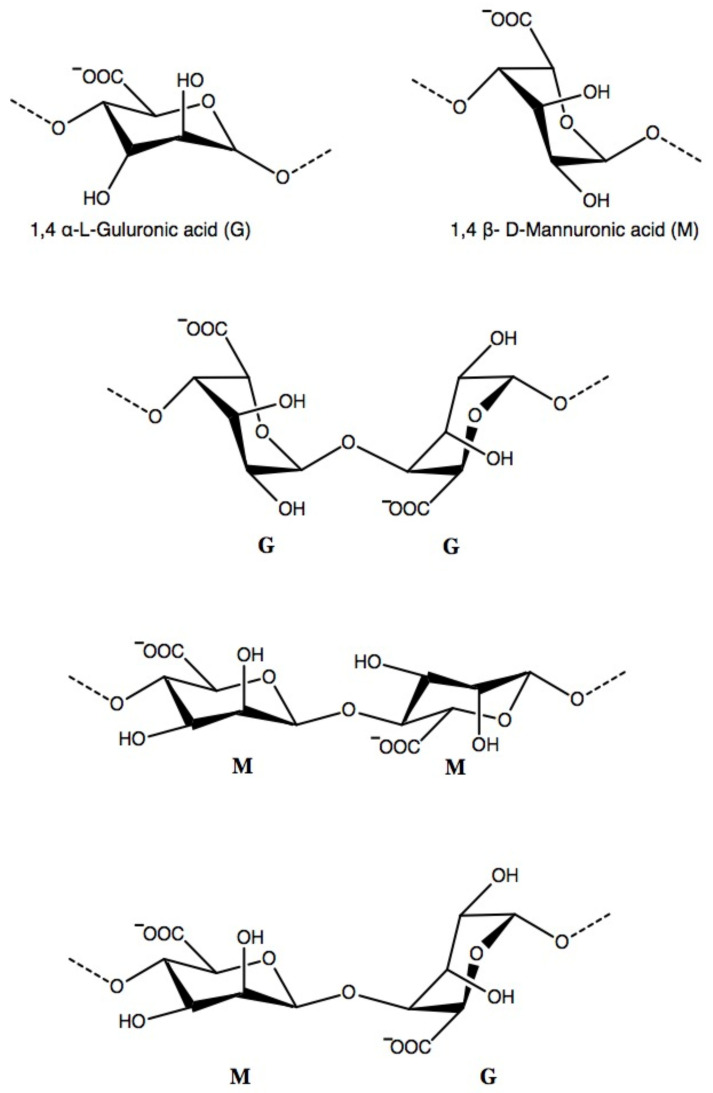
The chemical structure of 1,4 α-L-guluronic acid and β- D-mannuronic acid in alginate. The chemical arrangement of three block patterns, including homogenous monomers (-GGGG-, -MMMM-) and alternating monomers (-MGMGMG-) can be identified in alginate.

Alginate is a naturally occurring polysaccharide consisting of varying amounts of residues, including β-D-mannuronic acid (M) and 1–4 linked α-L-guluronic(G) [31]. Three different molecular structure patterns, including homogenous monomers (-GGGG-, -MMMM-) and alternating monomers (-MGMGMG-), are identified in alginates (Figure 2). M/G ratio and block length vary depending on the alginate source [32]. Up to now, over 200 alginate products are available in the market [32]. The mechanical properties of alginate are affected by the composition, length of the polymer chain, and molecular weight [33]. The molecular weight of alginate also varies with the sources. Increased proportion of G content in alginate and higher molecular weight can form strong mechanical properties.

In the market, the molecular weight of alginate varies from 33,000 to 400,000 g/mol [11]. Alginate with higher molecular weight also results in greater viscosity, contributing to the difficulty in processing [34]. Meanwhile, greater shear forces are generated, which can cause undesirable damage to the encapsulated cells and proteins [35].

#### 2.1.3. Biocompatibility and Biodegradability

Although many investigations on the biocompatibility of alginate had been undertaken in vitro and in vivo, a much-debated question about the effect of alginate composition on its biocompatibility had been discussed for many years. Until recently, it is widely accepted that alginate’s immunogenicity is associated with the presence of existing impurities, including heavy metals, endotoxins, and polyphenol [36]. Orive et al. demonstrated that poor biocompatibility, foreign body reaction, and inflammatory response of alginate primarily lie in the remaining impurities as they observed no immunological response when implanted highly purified alginate. Lee et al. further validated the importance of using purified alginate to reduce the risk of potential immunologic response by the host [37].

In human body, due to the lack of a specific enzyme named alginase to cleave alginate polymer chains, alginate is theoretically considered as nondegradable biomaterials. However, alginate cross-linked by covalent ions can be gradually dissolved by the ion-exchange reaction that continuous covalent ions release such as Ca^2+^ from alginate exchanges monovalent cations such as Na^+^ from the surroundings.

Mushollaeni et al. investigated the toxicity of alginate from two brown seaweed sources, including *Sargassum* and *Padina* by feeding Wistar mice with varying alginate concentrations. Alginate from *Sargassum* source ranging from 0.75% to 1% was regarded as safety concentration since the lower level of alanine aminotransferase (ALT) and aspartate transaminase (AST) was observed in the alginate-feeding group compared to the control group. However, alginate from *Padina* source could cause potential damage to the liver with a concentration above 0.75% [38].

#### 2.1.4. Ionic Cross-Linking

**Figure 3:**
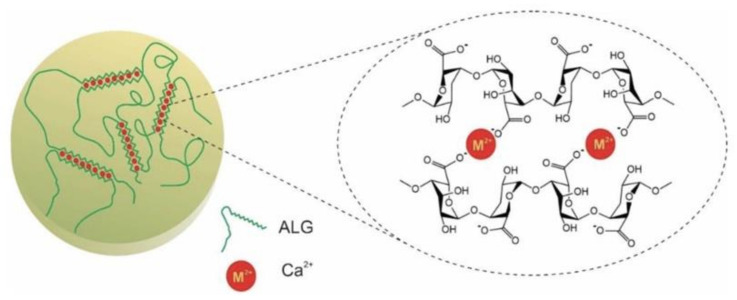
Schematic diagram of sodium alginate cross-linking by Ca^2+^. In the alginate containing −COO−group, Ca^2+^ exchanges with Na^+^ from guluronate block in ALG and interacts with separated ALG chains, forming the “egg-box structure”. Reproduced from [41] with permission. Copyright (2020) Springer Nature.

Alginate gelation can be achieved via physical and chemical methods. A 3D network of alginate is formed in the presence of divalent cation such as Ca^2+^, Sr^2+^, and Ba^2+^. Divalent cation can exchange Na^+^ from guluronate located in the separated ALG chains, forming the ‘egg-box’ structure (Figure 3) [39]. The most commonly utilized ion for alginate gelation is Ca^2+^ owing to its lower cytotoxicity effect, while it is reported that both Sr^2+^ and Ba^2+^-induced alginate is mechanically stronger than Ca^2+^-induced alginate. Gelation speed plays a critical role in controlling alginate gelation. Uniform hydrogel structure and superior mechanical integrity are achieved by decreasing the gelation speed, which is desirable for their application [40]. When directly using calcium chloride to initiate alginate gelation, it gives rise to uncontrollable and quick alginate gelling [40]. The reaction can be controlled by addition of carboxylate group and phosphate into calcium chloride solution since these ions can compete with Ca^2+^ to slow and delay the gelation reaction, finally forming a uniform hydrogel with greater mechanic integrity. In addition, temperature is considered another essential factor [40]. The cross-linking reaction can be slowed by low temperature as it reduces the reactivity of Ca^2+^. Therefore, it generates a uniform structure and greater mechanical integrity.

Gallium has been investigated as an ionic cross-linker for alginate gelation. Like calcium ion, gallium binds with the guluronate block in the separated ALG chains, forming a 3D structure [42]. It is worth noting that alginate-based hydrogel cross-linked by gallium possesses antibacterial function against Gram-positive and Gram-negative bacteria via the interpretation of metabolic pathway mediated by iron [43,44]. Rastin et al. employed 3D bioprinting technique with Gallium-induced ALG cross-linking together. Multilayered cell-laden alginate-methylcellulose hydrogel was fabricated using 3D bioprinting followed by gallium bath [45]. Moreover, there is no significant difference in encapsulated cell viability between Gallium and Ca^2+^-induced gelation group over a 7-day cell culture.

### 2.2. Collagen

Collagen is the major component protein found in the extracellular matrix in both hard and soft connective tissues. Collagen also has a dominant role in maintaining the structural and biological integrity of ECM [46]. Until now, 29 types of collagen have been identified in which triple-helical tertiary structure is commonly present. Collagen consists of three polypeptide chains, and each chain is comprised of repetitive Glycine-X-Y sequences where X and Y are often occupied by proline and hydroxyproline [47,48]. Glycine located at every third residue enables the tight packing of the three polypeptide chains into a tropocollagen molecule. The most common types of collagen are summarized in Table 1. In addition to its favorable properties, including excellent biocompatibility, weak immunogenicity, porosity, and degradability, collagen also has a vital role in regulating cell morphology, attachment, migration as well as differentiation [49,50]. Although various collagen types have been identified, fibril-forming collagens, including type I, II, III, V, and IV, have achieved the most popularity in tissue engineering. It is worth noting that type I collagen is considered a golden material in tissue engineering, which comprises up to 90% of protein found in connective tissue [51]. The sol-gel transition of collagen happened under defined conditions, such as neutral pH and physiological temperature [52].

#### 2.2.1. Collagen Source

Collagen can be obtained from various sources, including animal sources, synthetic sources, and marine sources. Type I collagen is the most used biomaterial in tissue engineering due to its widespread presence in animals and cost-effectiveness. Commercially available collagen in the market is mainly extracted from animals, including bovine, porcine, rat tail, and fish [53]. Yet, there is grave concern regarding the inflammation induced by remaining impurities in collagen, possible pathogen transmission, and batch-to-batch variability [54,55]. Indeed, it is reported that 2–4% of the population are allergic to collagen with a porcine and bovine source [56]. Recently, marine sources, including sponges and jellyfish, could be potential source candidates since there is no risk of pathogen transmission [55,57]. However, the poor thermal stability of marine-derived collagen may limit its application [58].

#### 2.2.2. Biodegradability and Immunogenicity

The majority of collagen degradation in vivo is digested by collagenase such as matrix metalloproteinases (MMPs). There are various collagenases in human body, and it is worth noting that collagen degradation rate varies in different collagenases and cross-linking methods. The collagen degradation via collagenase includes (1) matrix metalloproteinases binding to collagen first, (2) ability to unwind three polypeptides chain into a single chain since three-helical structure blocks the enzyme active sites, and (3) MMPs acting on each strand to cleave [59]. Different types of collagen can be digested by their corresponding MMPs [60,61,62]. After the degradation by MMPs, the remaining collagen fragments can be further degraded by gelatinase and other nonspecific enzymes. It is widely accepted that collagen exhibits excellent biocompatibility since it has a limited possibility to induce immunological response. Helical structure and telopeptide are regarded as the specific regions that can cause immune rejection [54]. Since collagen has low antigenicity, collagen-based material is widely used in tissue engineering and regeneration [63].

#### 2.2.3. Physical Cross-Linking

To date, there are various cross-linking methods for collagen including chemical cross-linking, physical cross-linking and enzyme-induced gelation. Although collagen cross-linked by chemicals including glutaraldehyde, carbodiimides, and polyepoxy compounds are more stable than other cross-linking methods, the cytotoxicity of the residuals and its byproducts after degeneration is a grave concern [64,65,66,67].

Collagen can be cross-linked by physical methods including ultraviolet (UV) light, gamma-ray radiation and dehydrothermal (DHT) [68]. Compared to chemical cross-linking, physical cross-linking is considered a begin method since it does not require cytotoxic chemicals; however, the low efficacy of physical gelation has been reported [69].

The DHT-induced collagen gelation is achieved by the formation of amide bonds between the free carboxyl group and amine group as a result of water removal under a higher temperature and vacuum condition [70]. In brief, the vacuum was set at 0.05 mbar and temperature at 40 degrees Celsius to remove water from COL molecule and prevent denaturation. After that, higher temperature (over 100 degree Celsius) was utilized to induce COL gelation further. It is worth noting that gelation and denaturation can co-occur. Collagen denaturation can occur under the higher temperature condition, finally resulting in poor-organized structure [71]. It is reported that temperature at 100 degree Celsius and 24 h treatment are the optimal parameters for DHT [72].

UV light is considered an effective and quick method compared to DHT treatment. Once collagen exposures to UV light, double bonds and aromatic rings can give rise to free radicals. The generated free radicals on the amino acids form covalent bonds between collagen molecules.

Collagen gelation via 254 nm UV light only takes 15 min, and UV-induced collagen shows higher resistance to degradation [73]. Dehydrothermal treatment is time-consuming as it requires a more extended period compared to UV light. DHT-induced collagen is more sensitive to trypsin but more resistant to degradation [74].

### 2.3. Collagen–Alginate Composite Hydrogel

#### 2.3.1. Stiffness and Cell-Binding Sites

The combination of collagen with alginate overcomes the limitation of each material and integrates their favorable properties altogether. The major hurdle that restricts the widespread application of collagen is its poor mechanical properties. Incorporation with other materials can improve the mechanical properties. It is reported that collagen in the combination with alginate not only enhances the mechanical properties of the composite hydrogel but also tunes the stiffness of CAC hydrogel simply via the concentration of Ca^2+^ [26]. It is worth noting that stiffness is of great importance in the determination of stem cell fate [75]. Scaffold with suitable and proper mechanical properties is capable of providing the mechanical cues to stem or progenitor cell towards certain cell lineages [76]. The increase in calcium concentration improves the stiffness of the composite hydrogel and vice versa.

**Figure 4:**
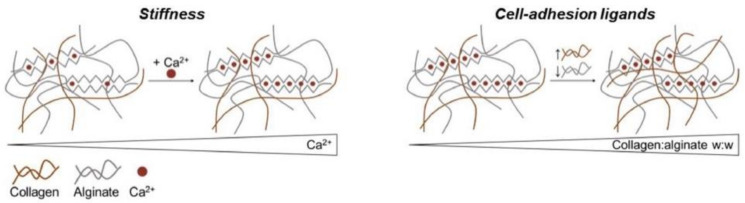
The relationship between the stiffness of composite hydrogel and calcium concentration and the association between collagen concentration and cell-binding sites inside the composite hydrogel. With the increase in Ca^2+^ concentration, the stiffness of composite hydrogel will be enhanced. Cell-adhesion ligands also increase as collagen concentration increases. Reproduced from [26] with permission, Copyright (2020) Elsevier.

On account of collagen containing cell-binding ligands and the inherent lack of cell-binding motifs inside alginate, the increase in the proportion of collagens in CAC hydrogel improves cell-binding ligands, which enable more cell adhesion and attachment, thereby maintaining cell viability and promoting cell proliferation. The relationship between the stiffness of composite hydrogel and calcium concentration and the association between collagen concentration and the cell-binding site inside the composite hydrogel are illustrated in Figure 4.

#### 2.3.2. Technique

Over the past decades, scientists have developed various techniques to manufacture CAC hydrogel on a large scale, surpassing the limitations of traditional 3D culture, which is time-consuming, technical, and laborious. In this section, 3D bioprinting on CAC hydrogel and other techniques will be discussed.

##### Three-Dimensional (3D) Bioprinting

Compared to traditional technique, 3D printing is an advanced technique that enables the construction of a complicated 3D structure via layer-by-layer manufacturing under the help of computer system [77]. 3D printing has achieved great success in the preparation of hydrogel scaffold in tissue engineering, especially in bone tissue and cartilage tissue engineering [78]. It is worth noting that 3D bioprinting enables the rapid fabrication of personalized implants which meet the individual need of patients [79]. The unique features of hydrogel include high water content and sensitivity to various stimuli including temperature, light as well as external signals. Therefore hydrogel is an excellent candidate in 3D bioprinting when working with cells [80].

3D printing can be classified into three categories including laser-based printing, nozzle-based printing and inkjet printer-based system [78]. Considering the good compatibility and ease of procession, alginate has received most popularity in bone tissue engineering as well as bioprinting [81,82]. Chondrocyte laden alginate hydrogel by 3D printing technique showed higher cell viability and promoted ECM deposition in vitro [83]. The main hurdle of using single materials as a bioink is poor mechanical properties [83]. Nevertheless, the poor mechanical properties of alginate usually result in instability of 3D structure, which is the main hurdle that restricts its application in hard tissue engineering [84]. Therefore, alginate in combination with other materials has received much popularity in 3D printing. Kim et al. demonstrated the prospect of a poly (ε-caprolactone) PCL/alginate scaffold with greater mechanical properties in hard tissue engineering since higher water absorption, greater retention efficacy of encapsulated cells, increased cell viability and significant calcium deposition were observed in PCL/alginate group compared to PCL group [85]. Recently, D’ Amora et al. combined 3D printing with surface modification, which enables the construction of 3D scaffold with structural and chemical gradients [86]. Moreover, recent advance also allows the incorporation of nanoparticle into the scaffold and the fabrication of injectable hydrogel [87]. Moreover, Collagen, one of the most popular bioinks, has received much research interest in tissue engineering [52].

Nozzle system is gaining most popularity in the preparation of scaffold-based hydrogel. The construction of 3D scaffold via nozzle system is based on three steps including (1) hydrogel solution was loaded into the system, (2) then it was forced out of the system into pre-designed mold, and (3) sol-gel transition. It is widely accepted that interlayer adhesion is essential for the successful fabrication of 3D structure. Besides, there are various factors which can play a dominant role in the fabrication including temperature, shear thinning, thixotropy and cross-linking methods.

A set of research groups proposed the use of dual concentric nozzle system to manufacture CAC hydrogel [88,89,90]. The dual concentric nozzle system consists of three different parts including outer syringe, inner syringe and CaCl_2_ bath. Collagen and alginate solution were loaded into inner syringe and outer syringe respectively to enable simultaneous injection of CAC solution into the calcium bath to form a fiber [88,89]. It is worth mentioning that fiber manufactured by dual concentric nozzle system can be molded into any irregular shapes. Besides, bioactive agents can be successfully loaded in collagen and alginate solution [89,90]. Buitrago et al. incorporated bioactive glass nanoparticle into alginate solution, which has demonstrated differentiation of stem cells towards osteogenic lineage [90].

Yao et al. adopted another electro-assisted inkjet printing to produce alginate microspheres coated with collagen [91]. Alginate microspheres were first fabricated via inkjet printing followed by collagen coating. The diameter of alginate-collagen microspheres, ranging from 245 μm to 657 μm, can be tuned by the following parameters including needle size, voltage, electrode distance and push speed.

In addition to 3D printing technique, there are other techniques to manufacture CAC hydrogel. One study applied a microconcave mold to fabricate CAC hydrogel for cell encapsulation, which overcame the traditional cell encapsulation technique [92]. In this study, islets cells were seeded into the microconcave mold followed by introduction of CAC solution. After that, CaCl_2_ solution was added to form islet spheroid. Microcapsules or spheroids can also be fabricated using microfluidics [93,94]. Jose et al. employed microfluidics to manufacture stem cell laden CAC microspheres. In brief, a CAC-stem cells mixture was loaded into the device. The CAC microspheres were fabricated when the solution passed through the inlet followed by shear force [94]. The techniques for the manufacture of CAC hydrogel are summarized in Table 2.

## 3. Application of CAC Hydrogel

### 3.1. Tissue Engineering

#### 3.1.1. Bone Tissue Engineering

In bone tissue engineering, despite transplantation of autografts into defects site is the gold standard, the limited autograft sources and potential complications such as the risk of bacterial infection and donor site morbidity cannot be ignored [95]. CAC hydrogel has found prospect as their potential in treating bone defects via delivery of stem cells or progenitor cell population, bone inductive growth factors, or the combination of cells and molecules.

Many studies have demonstrated that alginate hydrogel, compared to others, can be delivered into the human body in a minimally invasive way, its powerful molding ability that enables it fitting into any irregular defect sites and its tunning porosity to release many drugs to the defects [96,97]. Nevertheless, the biological property of alginate in terms of cell adhesion and attachment is limited. Therefore, alginate combined with other biodegradable biomaterials and RGD peptides in attempt to improve long-term cell viability and survival has attracted much research attention recently.

Collagen-based hydrogel is receiving much attention in bone tissue engineering since collagen is the major component protein found in bone tissue [98]. Despite collagen owns excellent biocompatibility and ECM-like structure that facilitates cell attachment and proliferation, the limited biomechanical stability, especially in the presence of cells and the poor moldability, limit its widespread application in the field of orthopedics [99,100,101,102]. What is more, it has been reported that collagen has limited ability to induce osteogenic differentiation from stem cells in comparison to other materials [99,100,101].

The composite hydrogel integrates the merits of each material and overcomes each limitation such as enhanced mechanical properties, powerful moldability, capability to maintain cell viability and potential to induce osteogenic lineage under defined environment. The excellent biocompatibility of CAC hydrogel has been extensively examined [90,103]. A study conducted by Benayahu et al. showed that up to six-month system stability of the composite hydrogel under different storage conditions in vitro and excellent biocompatibility by the observation of the absence of adverse effect and non-cytotoxicity near the implants [103].

In an attempt to accelerate bone regeneration, the ability of CAC hydrogel to deliver cell population to the defect site has also been examined. Perez et al. developed core-shell dual-layered hydrogels using a nozzle system in which cell-containing collagen and alginate were fabricated in the core and the outer shell respectively [88]. Moreover, the nozzle system enables hydrogel to be easily molded into any irregular shape, which is desirable and corresponds with clinical needs. More importantly, the authors illustrated that compared to the non-cell-loading construct, core-shell hydrogel loaded with bone marrow mesenchymal stem cells (bMSCs) exhibited considerable bone formation at the central region of the defect site in the rat model of calvarium defect [88]. CAC can also be incorporated with other biomaterials. Bendtsen et al. incorporated hydroxyapatite, one of the major component proteins found in bone, into CAC hydrogel [104]. The development of in situ injectable hydrogels provided a candidate scaffold for bone tissue engineering [104]. Moreover, CAC hydrogel containing chitosan (ChS) and hydroxyapatite (HAp) has shown their advantages for cell infiltration and proliferation [105].

**Figure 5:**
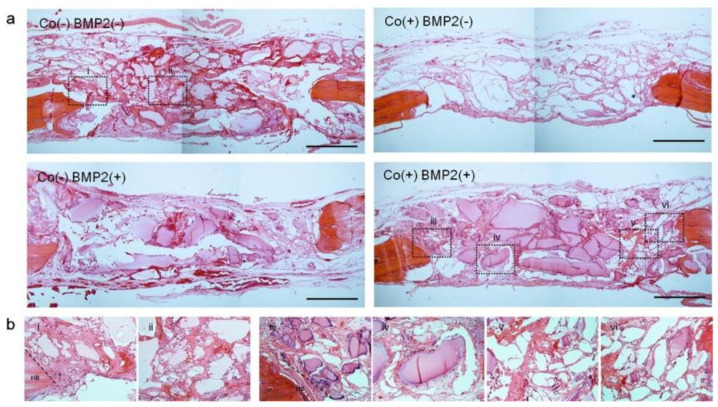
H&E stained sections of CAC implant in vivo after 6 weeks. (**a**) Low magnification image of CAC hydrogel groups including Co (−) BMP (−), Co (+) BMP (−) Co (−) BMP (+), and Co (+) BMP (+), (**b**) high magnification image of each groups. Reproduced from [89] with permission. Copyright (2015) Elsevier.

While CAC hydrogel can support bMSCs survival and proliferation, bMSCs osteogenesis happens in the presence of osteo-inductive molecules [104,106]. CAC hydrogel can be incorporated with other bioactive agents to provide mechanical cues to bMSCs. Perez et al. showed bMSCs differentiation towards osteocytes under the inductive condition in vitro [107]. CAC hydrogel is also being incorporated with novel nanoparticles to promote bone regeneration by guiding stem cell differentiation towards osteogenic lineages. The sequential delivery of cobalt ions followed by bone morphogenetic protein (BMP2) has shown promoting effect on bone regeneration in the rat model of calvarium defect (Figure 5). In particular, the author developed a core-shell CAC construct that enables the loading of Cobalt, which has a pro-angiogenic effect, and BMP-2 that stimulates bone regeneration into the outer shell and inner cone respectively. The scaffold promoted bone regeneration and angiogenesis [89]. Buitrago et al. further incorporated bioactive glass nanoparticles into the outer alginate shell using a nozzle system and showed that bioactive glass nanoparticles were capable of directing bMSCs cell differentiation into osteoblasts in vitro [90].

The delivery of bone morphogenetic proteins (BMPs) has also been explored in collagen–alginate hydrogel. Quinlan et al. showed the same kinetics BMPs release from Col-HAp scaffold incorporated with ALG microparticle [108]. HAp-Col-Alg scaffold with BMPs loading was also fabricated by Sotome et al., and the results showed the composite hydrogel is a potential drug carrier scaffold for bone tissue engineering [109].

#### 3.1.2. Cartilage Tissue Engineering

Due to its nonrenewable property, cartilage itself cannot regenerate once damaged. Therefore, cartilage regeneration poses a significant challenge in the field of orthopedics. Up to now, extensive efforts and investigations are focusing on developing alternative therapy to repair damaged cartilage. In particular, tissue engineering is considered a promising approach in cartilage regeneration.

The use of bMSCs shows great promise in cartilage regeneration as its potential differentiation into chondroblasts under certain conditions, and their favorable advantages. including ease of isolation and the limited autologous source [110]. Therefore, it is a pressing need to develop an ideal bMSC scaffold that highly recapitulates the native scenario and provides mechanical cues. Many studies have suggested that inducible molecules are essential for guiding chondrogenic differentiation of bMSCs [111]. Zhang et al. (2017) demonstrated that the CAC scaffold provides a suitable environment for bMSCs growth and proliferation. Moreover, bMSCs were capable of differentiating into chondrocytes by inducible agents secreted by co-cultured chondrocytes in vitro [111]. In addition, Ledo et al. (2020) successfully incorporated SOX9 polynucleotides, which play a vital role in chondrogenesis, into CAC hydrogel, which may provide new approach for cartilage tissue engineering [26].

**Figure 6:**
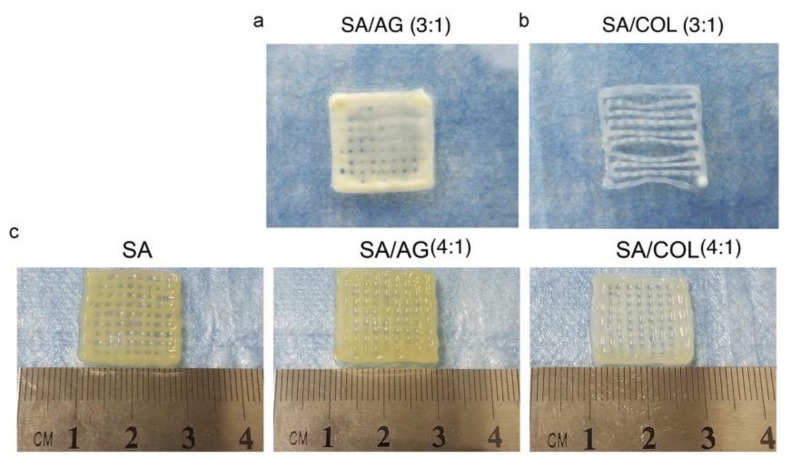
Small grids by 3D printing. (**a**) sodium alginate/agarose (3:1), (**b**) sodium alginate/collagen (3:1), and (**c**) sodium alginate, sodium alginate/agarose (4:1) and sodium alginate/collagen (4:1). Reproduced from [116] with permission. Copyright (2018) Elsevier.

**Figure 7:**
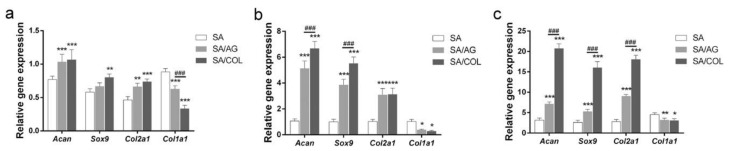
Expression level of genes including Acan, Sox9, Col2a1, and Col1a1 was determined by qRT-PCR. 3D constructs including SA, SA/AG and SA/Col in vitro after 3 days (**a**), 7 days (**b**), and 14 days (**c**). *, **, and *** indicated *p* < 0.05, *p* < 0.01 and *p* < 0.001 between SA/AG and SA/COL vs. SA. ### indicated *p* < 0.001 between SA/AG vs. SA/COL. Reproduced from [116] with permission. Copyright (2018) Elsevier.

Although transplantation of chondrocytes to damaged cartilage is attractive, one major issue in cartilage regeneration is the chondrocytes’ dedifferentiation characterized by the continuous loss of chondrocyte markers [112]. A set of studies have demonstrated that alginate is capable of dedifferentiating the differentiated chondrocytes [113,114]. Nevertheless, alginate hydrogel has limited ability to maintain chondrocyte viability, restricting the application in the preservation of chondrocyte phenotype. Collagen–alginate hydrogel has the potential to preserve chondrocytes phenotypes [115]. Jin et al. (2018) demonstrated that incorporating collagen into alginate can maintain long-term chondrocytes survival and viability while preserving chondrocyte phenotype. Although collagen gel showed higher cell viability when compared to CAC hydrogel, the enhanced chondrocyte dedifferentiation was observed. Incorporating collagen into alginate, providing a suitable environment for chondrocyte growth and proliferation, inhibits chondrocyte dedifferentiation and preserves its phenotype [115]. Yang et al. (2018) employed 3D printing to fabricate cartilage tissue using CAC hydrogel (Figure 6). In comparison with ALG and ALG/agarose group, CAC group showed higher chondrocyte survival and enhanced chondrocyte gene expression including Acan, Sox9 and Col2a1 and decreased Col1a1 gene expression which is a fibroblast cell marker (Figure 7). This study may provide new approach for cartilage tissue engineering [116].

#### 3.1.3. Intervertebral Disc (IVD)

Intervertebral discs lie between adjacent vertebras and function as cushions during movement, modulating spinal motion and providing flexibility [117]. An IVD consists of two distinct regions including annulus fibrous (AF) and nucleus pulposus (NP). Gelatinous nucleus pulposus (NP) is located in the inner region which is surrounded by fibrocartilaginous AF ring.

AF is a highly organized tissue composing of collagen (type I) and proteoglycans, while NP is an unaligned structure consisting of collagen (type II) and proteoglycans [118]. Low back pain is highly associated with IVD degeneration. The majority of patients with IVD degeneration suffer from low back pain, which poses a significant burden on their quality of life. Current treatment for low back pain includes analgesics, anti-inflammatory and surgical intervention. Spinal fusion is regarded as gold standard for treating low back pain caused by IVD degeneration. However, it comes with limited spinal mobility and increased chance of adjacent IVD degeneration [119].Therefore, many efforts and strategies have been placed on the development of engineered IVD to replace the degenerated IVD [120,121,122]. In particular, hydrogels including alginate, chitosan, hyaluronic acid, and collagen have been found to have potential as a scaffold in the IVD degeneration [121,123].

Collagen-based hydrogels have been widely investigated for their potential scaffold to treat IVD regeneration. Moriguchi et al. seeded AF cells into high-density collagen gel and showed that cell-loading group has the capacity of promoting IVD regeneration compared to acellular group in the rat model of IVD degeneration induced by needle puncture [124]. Hussain et al. fabricated MSCs laden high-density collagen hydrogel and showed their prospect in AF and NP regeneration in a sheep model of lumbar IVD degeneration [125].

Both alginate and collagen have been widely investigated in IVD regeneration. Tsaryk et al. engineered collagen–alginate IVD construct containing gelatin microspheres. Moreover, the composite hydrogel supported MSC cell survival and promoted its differentiation towards chondrocytes [126]. Bowles et al. seeded AF cells into composite hydrogel in which collagen and alginate were fabricated in the outer region and inner region respectively. The increased alignments of circumferential fibril were observed in the 1% collagen group in vitro [127], which provide a new approach for the engineered-IVD with more complicated structure. Gloria et al. employed 3D bioprinting to manufacture IVD construct containing PCL, collagen with low molecular weight, HA and chitosan nanoparticles [128]. The study further demonstrated that the injectability of this scaffold, similarity of compressive modulus to the natural IVD, and maintenance of cell viability in vitro [128]. Moriguchi et al. constructed engineered IVD construct consisting of outer collagen hydrogel and inner alginate hydrogel [129]. The author also seeded AF and NP cells into both inner and outer region [129]. The implantation of the designed interverbal discs into the canine model of discectomy showed a successful integration of composite hydrogel into the host tissue with no immune response [129].

### 3.2. Tissue Regeneration

CAC hydrogel is also being extensively explored for the potential in the field of tissue regeneration to generate a range of tissues, including vessel, neuron, liver, and vocal folds owing to its favorable properties, including gelling under mild condition, proper environment for cell growth and proliferation, and ease of incorporation of bioactive agents, and greater retention of bioactive agents.

#### 3.2.1. Blood Vessel

In the human body, blood vessels play an important role in regulating and maintaining homeostasis, such as carrying oxygen and transporting nutrients to tissues, clearance of metabolic waste from cells, and delivering stem cells or progenitor cells to the tissues [130].

The establishment of the blood vessel network, defined by new blood vessel formation from existing blood vessels via cell proliferation, sprouting, and migration, is of great importance in the field of tissue engineering, especially in 3D constructs on a large scale since angiogenesis is crucial for improving transplanted cell survival, and normal function of newly constructed tissues [130,131]. Cells could suffer from hypoxia and receive limited nutrient supply once the distance between cells and blood vessels is over a few hundred microns (150–200 μm) [132]. The situation is even worse in 3D constructs as cells in the core receive oxygen and nutrients only via a blood perfusion reaction rather than a diffusion pathway [133]. In addition, neovascularization is also holding great promise to treat patients with myocardial infarction and stroke.

In order to achieve neovascularization, there are several strategies, including the delivery of pro-angiogenic cells to the body, transportation of angiogenic stimulating factors such as vascular endothelial growth factor (VEGF) and platelet-derived growth factor (PDGF), or the combination of both strategies. However, the direct delivery of cell suspension or angiogenic growth factors to the ischemic tissue comes with poor retention of pro-angiogenic cells and therapeutic factors [134]. In order to combat this issue, many efforts have been placed on achieving better retention of cells and therapeutic agents using biocompatible hydrogel in recent years.

**Figure 8:**
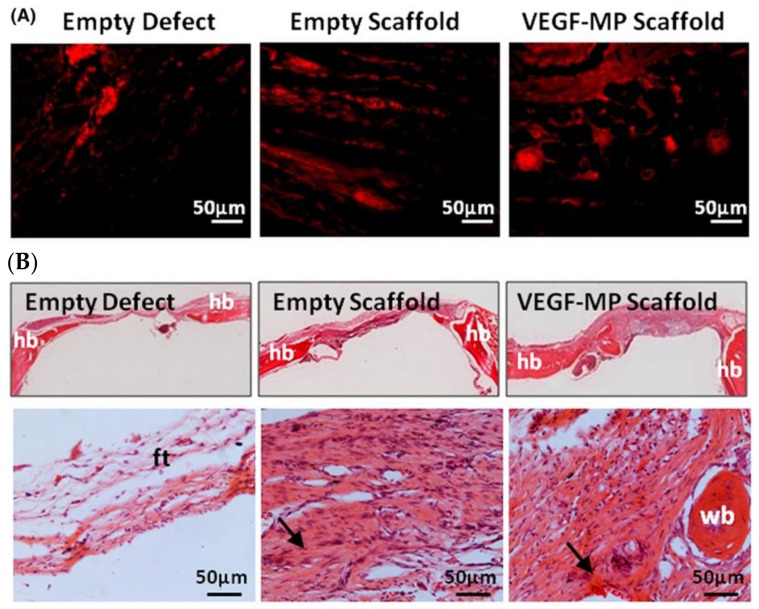
Immunobiological images of blood vessel formation and H&E sections. (**A**) Immunological images of endothelium labeled by CD31 (red). (**B**) H&E staining of empty defect, empty scaffold and VEGF-MP scaffold. HB, FT and WB for host bone, fibrotic tissue, and woven bone. Arrows indicated new bone formation. Reproduced from [135] with permission Copyright (2015) John Wiley & Sons, Ltd. Hoboken, NJ, USA.

CAC hydrogel has been studied as a drug delivery platform to deliver several angiogenic growth factors to the ischemic tissues. Quinlan et al. incorporated collagen-based scaffold with spray-dried alginate microparticle to achieve long-term (at least 35 days) release of VEGF in vitro [135]. More importantly, when implanted into a rat model of calvarial bone defect, the functionalized composite scaffold was capable of promoting bone regeneration and stimulating new blood vessel formation as demonstrated by threefold increase in the number of endothelial cells labelled by CD31 in comparison to the empty scaffold group (Figure 8). Ali et al. engineered alginate collagen microspheres loaded with FGF-2 and showed at least a 7-day FGF-2 release [136]. The direct transplantation of injectable spheres into ischemic tissue enhances blood vessel formation in zebrafish [136].

#### 3.2.2. Neuron

In comparison with other tissues, the extracellular matrix of the brain is considered complicated and soft with a heterogenous network of proteoglycan [137]. Hyaluronic acid, a mucopolysaccharide, interacted with other proteins to contribute the majority of proteoglycan [138]. Nevertheless, the cross-linking of hyaluronic acid-based hydrogel requires chemical modification or physical methods including high temperature, base and UV [139]. The cytotoxicity of the chemical cross-linker or the uncompromised physical method limits its use when working with cell [139]. Alginate is receiving research interest in neuron regeneration in light of its high structure similarity to hyaluronic acid and gelling method under mild condition [140]. Its incorporation with collagen, which contains cell binding motifs to form CAC hydrogel has also been investigated for its ability to encourage neurogenesis.

Moxon et al. demonstrated that compared to alginate hydrogel, composite hydrogel provides a suitable 3D environment that maintains encapsulated cells survival, stimulates neuronal maturation and encourages the projection from neurites as demonstrated by the significant expression of synaptophysin [25]. In addition to collagen fibrils that provide mechanical cues to iPSC-derived neuron, the study further demonstrated that increased stiffness via Ca^2+^ concentration significantly decreased the expression of microtubule associated protein 2 which is a marker for neurogenesis. The composite hydrogel may be a potential approach to treat patients with disorders related to damaged or degenerated nervous tissue as the work by Jose et al. demonstrated that neural stem cell encapsulated into CAC microsphere using microfluidics technology maintained at least 10-day cell viability and differentiated into neural lineage in the ex vivo model of spinal cord injury [94]. CAC hydrogel has also been combined with other biomaterials and peptides to promote neuronal differentiation. Collagen–alginate composite hydrogel containing hyaluronic acid and methacrylic anhydride has triggered the neuronal differentiation from iPSCs [141].

#### 3.2.3. Liver

Collagen–alginate hydrogel may be a promising approach for the replacement of damaged liver tissue. Chan et al. successfully employed microfluidics technology to encapsulate hepatocytes into CAC hydrogel [93]. Moreover, hepatocytes showed greater functions [93]. In particular, when compared to the control groups including collagen and alginate group, the total albumin secretion from hepatocytes in the CAC group was significantly higher in vitro. In addition, increased cytochrome enzyme activity was observed in the CAC group.

#### 3.2.4. Vocal Folds Restoration

The work conducted by Hahn et al. showed promise as a potential scaffold in vocal fold restoration [142]. A significant increase in synthesized reticular collagen fibrils was observed in CAC group compared to the collagen-hyaluronic acid group. In addition, CAC hydrogel maintained excellent mass and no reduction in gel size over a 42-day cell culture, further suggesting that CAC hydrogel exhibited a desirable 3D matrix for vocal folds regeneration compared to the collagen-HA scaffold.

CAC hydrogel has been found great potential as a scaffold in tissue engineering and tissue regeneration. Table 3 provides a list of existing studies of CAC hydrogel as a scaffold that can be used in different tissues.

### 3.3. Biomedical Sciences

#### 3.3.1. Wound Dressing

The definition of a wound is the laceration or discontinuity of epithelium or mucosa resulting from violence, accidents or surgical procedure. Wound can be classified into two categories, including acute and chronic wound, based on the duration and nature of the healing process [143,144]. Wound healing is a complicated, intricate, and dynamic process orchestrated by an array of cells, growth factors, cytokines, and ECM, which requires an appropriate and suitable environment to assist the healing process [145]. The wound dressing is of great importance during the healing process since a proper wound dressing can accelerate the physiological reconstruction meanwhile minimizing the risk of bacterial infection and protecting wound contamination from the surroundings and retention of a moist environment for the wound [146]. The ultimate goal of wound care is to completely restore and reconstruct the damaged tissue to its prior functional level with satisfactory cosmetic results [147].

When designing an ideal wound dressing, various considerations and factors should be taken into account, including its ability to (1) keep and maintain a moist environment, (2) promote new blood vessel and physiological reconstruction of connective tissue and skins, (3) accelerate epidermal migration (4) permit gas exchange between injury site and the surroundings (5) remove easily (6) fight microbial infection (7) maintain appropriate environments such as PH and temperature and (8) offer sterilized condition with noncytotoxic and nonallergenic properties [148].

Traditional wound dressings, including gauze, and bandages, mainly provide a physiological barrier to prevent microorganism infection, meanwhile permitting exudate adsorption to maintain a dry environment for the wound site [149]. Nowadays, the market of modern wound dressing is expanding as it can provide a moist environment that stimulates cell proliferation and differentiation, therefore accelerating the healing process and minimizing the chance of bacterial infection compared with traditional wound dressing. There are growing modern wound dressing products available in the market that combine two or three natural biomaterials, including alginate, collagen, chitosan, and cellulose, since the composite hydrogel enables the integration of each individual favorable properties to meet the requirements of ideal wound dressings.

Alginate-based wound dressing has been applied as one type of modern dressings for many years since alginate provides several superior features, including biocompatibility, low cytotoxicity to mammalian cells, and moist environment retention compared to traditional ones [149]. Plenty of studies have reported that alginate exhibited a great promoting effect on the wound healing process. However, the poor hemostatic effect has also been recognized. Collagen has been studied as a biomaterial in wound dressing owing to its unique characteristics, such as excellent biocompatibility and biodegradability [150]. Collagen has been found to accelerate wound healing by increasing cell proliferation, guiding cell differentiation, promoting neovascularization, and accelerating blood coagulation [151]. Incorporating collagen with alginate has attracted much research attention recently due to its properties, including excellent biocompatibility, low immunogenicity and superb hemostatic effect. In addition to that, collagen–alginate wound dressings have exhibited unique properties, including (1) alginate could provide a moist environment which is essential and beneficial for wound healing, (2) collagen can stimulate fibroblast migration, promote re-epithelization and angiogenesis, (3) mild gelling that enables cell encapsulation and (4) ease of incorporation with other materials and antimicrobial agents (Table 4). Therefore, CAC composite hydrogel is a potential candidate in wound care. Several collagen–alginate wound dressing products, including FIBRACOL Plus, DermaCol Ag, and ColActive Plus, are commercially available.

Recently, a novel wound dressing comprised of two or three biomaterials such as collagen, alginate, chitosan, HA, and cellulose incorporated with functional and bioactive agents has been studied since single material cannot meet all requirements of the ideal wound dressing [160].

Carl et al. demonstrated that, compared to traditional dressing, the collagen–alginate wound dressing consisting of 90% collagen and 10% alginate presented a decreased wound healing duration and reduced chance of infection and contamination, improved patients’ compliance, and higher satisfaction in patients with chemical matricectomies [154]. In another randomized clinical trial, patients with diabetic foot ulcers were treated with traditional wound dressing (gauze moistened with 0.9% NaCl) and a modern collagen–alginate wound dressing. The results showed that a reduction of wound size in the collagen–alginate group compared to the gauze group. More patients reported having complete wound healing in the CAC group, which suggested that the collagen–alginate wound dressing has more efficacy than commercially used gauze wound dressing [155]. Moreover, greater satisfaction was shown in collagen–alginate treatment group owing to its ease of removal and limited wound care duration. Collagen–alginate dressing group showed significantly less painful index, cost-effectiveness, decreased duration of re-epithelization. The transplantation of CAC scaffold into defects promoted wound healing in vivo. Collagen–alginate wound dressings have more advantageous effects on promoting wound healing [103].

The use of stem cells in wound dressing has emerged as a promising strategy due to their potential to reconstruct tissue to a pre-injured state. A recent study by Zhang et al. demonstrated that encapsulated human umbilical cord mesenchymal stem cells (HUCMSCs) in collagen–alginate hydrogel maintained good viability and growth factors secreted by HUCMSCs inhibited immunological response, thereby promoting wound healing and tissue reconstruction [159].

CAC hydrogel can also be incorporated with other biomaterials to gain more favorable properties. A recent study by Xie et al. demonstrated that coupling chitosan, which exhibits non-cytotoxicity, antibacterial effect, hemostatic effect, and biocompatibility, into CAC wound dressing facilitated platelet aggregation and formation of a fibrin clot, accelerated cell migration including fibroblasts and endothelium to the wound site, thereby promoting wound healing in the rat model of the full-thickness wound [157]. The CCA wound dressing was further attached to polyurethanes (PU) membrane to obtain seawater resistance [157]. Patients with acute full-thickness wounds suffered from a more extended wound healing period, which increases the risk of bacterial infection, finally leading to wound ulceration, necrosis, and even life-threatening complications. It is estimated that over two million people carry wounds containing antibiotic-resistant bacteria contamination. The growing prevalence of multidrug-resistant (MDR) pathogen has led to more than twenty thousand deaths yearly, providing a strong impetus to develop modern dressing with anti-bacterial infection [161]. Up to date, the functionalization of wound dressings with antiseptic and anti-inflammatory agents are showing a more powerful effect on promoting wound healing [162]. CAC wound dressing does not possess antibacterial properties. Therefore, research into the development of novel dressing designs with antimicrobial properties by incorporating nanoparticles or antiseptic agents has received much attention. Feng et al. incorporated polymyxin B sulfate and bacitracin into CAC wound dressing. They reported that CAC-PB exhibited an excellent antibacterial effect against *E. coli* and *S. aureus* and promoted wound healing in a rat model of a full-thickness wound as the evidence of observation of granulation tissue, the new formation of the vascular network, and re-epithelization [156]. Silver has been extensively studied as its excellent antimicrobial activity against fungi, bacteria, and viruses. In a recent study by Zhang et al., AgNps, a silver nanoparticle, was introduced into a collagen–alginate wound dressing to achieve the anti-microorganism function [158]. Functionalization of CAC with AgNps showed their significant antibacterial effect against *E. coli* and *S. aureus*. Nevertheless, the dose-dependent cytotoxicity of silver nanoparticles to mammalian cells and impaired wound healing process cannot be overlooked. Therefore, scientists are searching for novel wound dressing with broad antibacterial function and weak toxicity to mammalian cells.

Antimicrobial peptides (AMPs), which belong to one part of innate immune system, have found their benefit for promoting wound healing and therapeutic potentials to treat wound infection on account of their broad-spectrum antimicrobial effect on both Gram-positive and Gram-negative bacteria and inherent favorable properties including limited cytotoxicity to human mammalian cells and regulatory effect on immune cell migration and dendritic differentiation [163]. Lin et al. conjugated AMP Tet213 with CAC hydrogel containing hyaluronic acid via the chemical bond. Moreover, slow and controlled release of AMP Tet213 from Alg/Ha/Col-AMP wound dressing showed a significant inhibitory effect on bacterial infection such as *E. coli* and *S. aureus* and promoting effect on wound healing in vivo via its regulatory role in inflammation, collagen deposition, and angiogenesis [153].

Although a vast amount of biomaterial-based wound dressings has been developed, there is limited knowledge regarding the impact of biomaterial scaffold on the wound healing process. One study conducted by Cunha et al. synthesized a set of CAC scaffolds with different storage modulus via tuning the concentration of Ca^2+^. The result demonstrated that varying stiffnesses caused the morphologic change on fibroblasts and regulated the secretion of inflammatory factors, therefore accelerating or inhibiting the wound dressing process [164].

#### 3.3.2. Encapsulated Cell Therapy

CAC hydrogel has been developed as cell carriers to deliver bioactive agents secreted by encapsulated cells inside the device to treat a set of diseases including type 1 diabetes and retina degeneration.

The concept of ECT is to deliver fresh and synthesized therapeutic agents secreted by live cells either from allogeneic or xenogeneic sources that are encapsulated in a semipermeable membrane. The semipermeable membrane allows gas exchange and nutrients supply while avoiding immunological reaction from host upon transplantation via blocking the immune mediators [165]. Moreover, the function of polymers is to provide mechanical protection to the enclosed cells from shear force damage [166]. The history of cell encapsulation can be traced back to the early 1930s when Bisceglie et al. encapsulated cancer cells into the polymer scaffold and successfully transplanted the capsule into pig abdomen. The concept of “artificial cell” was firstly depicted by Tomas Chang, who subsequently proposed the idea of encapsulated cell therapy [167].

Various preclinical and clinical trials on the efficacy of ECT to treat central nervous system degeneration and retinal degeneration, including amyotrophic lateral sclerosis, Huntington’s disease, Alzheimer’s disease as well as retinitis pigmentosa have been undertaken [168]. There are plenty of biomaterials which have been investigated and developed as a scaffold for ECT device. Among these biomaterials, alginate is the most commonly used biomaterial either alone or in combination with other biomaterials. Nevertheless, the inherent lack of cell-binding sites limits its widespread application in ECT. Therefore, incorporating collagen, which contains cell-binding motifs, maintains cell viability and promotes cell proliferation via facilitating cell adhesion and attachment. CAC hydrogel is becoming the focus in ECT as composite hydrogel combines collagen and alginate merits. In this section, encapsulation of glial cell line-derived neurotrophic factor (GDNF)-secreting human embryonic kidney (HEK) cells, islet cells as an alternative therapy to treat retinal degenerative disease, and type 1 diabetes will be illustrated.

##### Islet Cells

The increasing number of diabetes mellitus (DM) imposes a significant burden on the healthcare system around the world. Pancreatic islets transplantation recently has been considered as a potential treatment for DM patients. Nevertheless, there remains the difficulty in the isolation of pancreatic islet cells from the donor, maintaining viability during the procedure, and the relative risk of pancreas transplantation is of grave concern. The work conducted by Lee et al. revealed the great promise of ECT as a treatment for type 1 diabetes. The author encapsulated islet cells into collagen–alginate composite hydrogel using the concave well array for manufacturing CAC hydrogel on a large scale.

The author showed the successful encapsulation of islet spheroids into CAC hydrogel. Compared to the pure alginate group, CAC showed increased islet cell viability and integrity for at least eight weeks, suggesting CAC is an excellent biomaterial scaffold for maintaining islet cell viability and function [92]. Moreover, the incorporation of collagen enhanced the inward diffusion of oxygen and nutrient to the islet cells. More importantly, encapsulated islet spheroids showed a glucose-controlling effect compared to the direct implantation of intact islets into the mice with diabetes. Direct implant of intact islets demonstrated robust and significant CD 45 fluorescent signals compared to the encapsulated islet group, strongly suggesting that CAC protected islets cells from immune attack and provided mechanical protection.

##### GDNF-Secreting HEK Cells

GDNF has demonstrated a neuroprotective effect on both the central nervous system (CNS) and peripheral nervous system (PNS). GDNF has been proved its neuroprotective effect in a set of animal models. Recently, GDNF has been applied into preclinical and clinic trials to treat CNS degeneration. Nevertheless, considering the relative half-life of GDNF, and the potential adverse effect of systemically administration of GDNF, ECT that enables continuous GDNF release is required. Wong et al. encapsulated GDNF-secreting HEK cells into three-dimensional collagen microspheres to achieve the continuous GDNF secretion. When enclosing GDNF-secreting HEK cells into 3D collagen microsphere, it showed dramatically increased GDNF secretion compared to monolayer cell culture since collagen recapitulates a native tissue-like environment for cells to grow and proliferate [169]. Further analysis suggested that GDNF secreted from 3D collagen microspheres possesses bioactive properties as GDNF promoted neurite extension, highly suggesting the potential of cell-collagen encapsulation as a drug delivery platform in the field of pharmaceutics.

Another study conducted by the same group introduced alginate into collagen-cell microspheres to limit cell migration from collagen scaffold to the surroundings for further application since cell migration from ECT may elicit immunological response and immune rejection [170]. In this work, the author illustrated that the collaboration of alginate with collagen is able to prevent cell migration and also allow continuous GDNF secretion from the device. Moreover, cell viability is highly associated with alginate concentration. In summary, the work developed a novel CAC ECT device as a potential drug delivery platform to treat CNS degenerative disease. Wong et al. further developed CAC ECT device from microsphere to cylinder-like structure as a potential treatment for retinal degeneration and possibly retinitis pigmentosa. The work demonstrated that long-term system stability and performance of CAC ECT design can be tuned by alginate concentration and the importance of initial cell density on the optimization of ECT device. In vivo studies demonstrated that rat vitreous can support cell survival and proliferation inside CAC ECT device and GDNF secretion from the device [22]. Wong et al. further demonstrated that the performance of CAC ECT device could be tuned by varying parameters, including initial cell density, gelation sequence, alginate molecular weight, and concentration. More importantly, continuous GDNF secretion from the CAC ECT device has a neuroprotective effect on photoreceptor both histologically and electrophysiologically in the rat model of photoreceptor degeneration, which strongly implied that CAC ECT platform is a promising treatment to treat a group of retinal degenerative diseases [23].

#### 3.3.3. Tumor Biology

There is no doubt that cells live in the extracellular matrix that facilitates cell interaction, cell migration and cell attachment to basement, allows metabolic waste exchange and provides oxygen and nutrients to the cells. Although the vast majority of research into cancer studies are performed in two-dimensional (2D) cell culture, the limitation of 2D cancer cell studies compared to 3D culture cannot be ignored, including lack of cell interaction to ECM and cell migration, limited signal transduction, and the absence of cytoskeletal organization, which may lead to inconsistent findings between in vitro and in vivo studies.

The proliferation of tumor cells and their morphology are associated with the stiffness of 3D scaffold. A study showed that higher 3D stiffness promoted multicellular aggregate proliferation, which may provide an alternative strategy for cancer treatment and diagnosis [171]. Wang et al. further demonstrated the effect of stiffness on tumor cell migration. The study showed that higher stiffness can decrease cell volume, reducing the tumor cell migration capability [172]. Liu et al. also developed a tumor-like scaffold to mimick in vivo tumor scenario and showed tumor invasion in the 3D scaffold is similar to in vivo environment [173].

## 4. Conclusions and Future Perspectives

CAC hydrogel has shown its great potential in tissue engineering and biomedical sciences owing to its wide availability, excellent biocompatibility, ease of gelation, and weak cytotoxicity. The unique properties of CAC hydrogel include enhanced mechanical properties, moldability, and tunning porosity. Besides, CAC can also be incorporated with other materials, molecules, and nanoparticles to obtain more favorable features. In particular, in tissue engineering, CAC has been developed as a platform to deliver molecules or cells to the defect site. Moreover, Composite hydrogel can be further incorporated with other bioactive agents, especially in bone tissue engineering. CAC hydrogel is also being extensively explored for its potential in tissue regeneration, including angiogenesis, liver, neuron, and vocal folds. CAC hydrogel promotes wound healing by providing a moist environment and cell-binding site that enables cell migration and cell proliferation. The ease of incorporation of antibacterial agents and AMPs further expands their application in wound care. CAC hydrogel has been investigated as a drug delivery platform in the field of ECT. ECT device can be optimized by different parameters such as initial cell density, ALG concentration and molecular weight, and collagen concentration, which provides a new view for ECT application.

However, the majority of CAC studies in cartilage tissue engineering are performed in vitro. The applicability of CAC in cartilage regeneration warrants further animal investigation. 3D printing may provide an alternative approach for CAC application in cartilage tissue engineering. Although hydrogel has been investigated in IVD regeneration, the construction of IVD for clinical use is still a considerable challenge. More investigations should be placed on the generation of engineered-IVD with optimal mechanical properties, the integration of engineered-IVD to host tissue, and the maintenances of encapsulated cell viability. The performance of CAC ECT device can be tuned via initial cell number, ALG and COL concentration, and molecular weight. Therefore, the efficacy of ECT in the treatment of diseases requires more investigation. One of the merits of composite hydrogel is its tunning mechanical properties. However, there are limited studies regarding the effect of stiffness on encapsulated cells and tumor cells, which warrants more investigation.

## Figures and Tables

**Table 1:** Collagen types, molecular formula, and tissue distribution. Reproduced from [46] with permission. Copyright (2010) MDPI AG.

	Type	Molecular Formula	Polymerized Form	Tissue Distribution
Fibril-Forming (fibrillar)	I	[α1(I)]2α2(I)	fibril	bone, skin, tendons, ligaments, cornea (represent 90% of total collagen of the human body)
	II	[α1(II)]	fibril	cartilage, intervertebral disc, notochord, vitreous humor in the eye
	III	[α1(III)]3	fibril	skin, blood vessels
	V	[α1(V)]2α2(V) and α1(V)α2(V)α3(V)	fibril (assemble with type I)	*idem* as type I
	XI	α1(XI)α2(XI)α3(XI)	fibril (assemble with type II)	*idem* as type II
Fibril-associated	IX	α1(IX)α2(IX)α3(IX)	lateral association with type II fibril	cartilage
	XII	[α1(XII)]3	lateral association with type I fibril	tendons, ligaments
Network-forming	IV	[α1(IV)]2α2(IV)	sheet-like network	basal lamina
	VII	[α1(VII)]3	anchoring	beneath stratified squamous epithelia

**Table 2:** Summary of novel techniques for the manufacture of CAC hydrogel.

Technique	Description	Incorporation of Bioactive Agent	Cell Type	Hydrogel Characteristics	Reference
Dual concentric nozzle system	Alginate and collagen solution were loaded into outer and inner syringe respectively. Simultaneous injection of ALG and COL solution into CaCl_2_ bath to form a fiber	Bioactive glass nanoparticles loaded in the shell [90]	Mesenchymal stem cells (MSCs)	CAC fiber (core diameter: 700–1000 μm and shell diameter: 200–500 μm) [88]	[88,89,90]
		BMP loaded in the core and cobalt loaded in the shell [89]		CAC fiber (core diameter: 1000 μm and shell diameter: 150 μm [89]	
Electro-assisted inkjet printing	Alginate microsphere was fabricated by inkjet printing followed by collagen coating	Nil	Endothelial cell	CAC microsphere	[91]
Concave well mold	CAC containing islet cells were introduced into concave well mold followed by Ca^2+^ bath	Nil	Pancreatic islets cell	Islets spheroid	[92]
Microfluidics	CAC microspheres/spheroids were generated when pass through the inlet followed by shear force	Nil	Stem cell	CAC microsphere	[93]
		Nil	Liver cell	CAC spheroid	[94]

**Table 3:** Summary of CAC as a scaffold in tissue engineering and tissue regeneration.

Tissue Engineering or Tissue Regeneration	Tissue Application	Reference
Tissue engineering	Bone	[88,89,90,104,105,109]
	Cartilage	[26,115,116]
	IVD	[129]
Tissue regeneration	Blood vessel	[135,136]
	Neuron	[25,94,141]
	Liver	[93]
	Vocal folds	[142]

**Table 4:** Summary of CAC hydrogel in wound dressing.

Composition	Finding	Reference
Collagen–alginate-AMPs	Antimicrobial activity and no cytotoxicity to fibroblasts	[152]
Collagen–alginate-hyaluronic acid-AMPs	Antimicrobial activity, good biocompatibility, reduced inflammation, and angiogenesis	[153]
Collagen–alginate wound dressing	Accelerating wound healing and great patients’ satisfaction	[154,155]
Aminated collagen-oxidized alginate-polymyxin B sulfate-bacitracin	Antimicrobial activity, angiogenesis, and reepithelization	[156]
Chitosan-collagen–alginate dressing-PU membrane	Hemocompatibility, reepithelization, and water-resistance	[157]
Collagen–alginate-AgNPs	Antibacterial activity but dose-dependent cytotoxicity to mammalian cells	[158]
Collagen–alginate-hUCMSCs	Promote wound healing, reepithelization, and angiogenesis	[159]

## Data Availability

Not applicable.
